# The protein tyrosine kinase inhibitor, Genistein, delays intervertebral disc degeneration in rats by inhibiting the p38 pathway-mediated inflammatory response

**DOI:** 10.18632/aging.102743

**Published:** 2020-02-05

**Authors:** Jun Ge, Quan Zhou, Xiaoqiang Cheng, Jiale Qian, Qi Yan, Cenhao Wu, Yufeng Chen, Huilin Yang, Jun Zou

**Affiliations:** 1 Department of Orthopaedic Surgery, The First Affiliated Hospital of Soochow University, Suzhou, Jiangsu 215006, China; 2 Department of Orthopaedic Surgery, The Affiliated Huai'an Hospital of Xuzhou Medical University, Huai’an, Jiangsu 223002, China

**Keywords:** p38 MAPK pathway, intervertebral disc degeneration, Genistein, phosphorylation

## Abstract

The treatment for intervertebral disc degeneration (IDD) has drawn great attention and recent studies have revealed that the p38 MAPK pathway is a potential therapeutic target for delaying the degeneration of intervertebral discs. In this study, we analyzed a nature-derived protein tyrosine kinase inhibitor, Genistein, and its function in delaying IDD in rats both *in vitro* and *in vivo* via the p38 MAPK pathway. Nucleus pulposus cells treated with Genistein showed better function compared with untreated cells. Further study revealed that Genistein could play a protective role in IDD by inhibiting phosphorylation of p38, consequently inhibiting the p38 pathway-mediated inflammatory response. The rat IDD model also demonstrated that Genistein could effectively delay the degeneration of intervertebral disc tissue. The current study reveals new biological functions of Genistein, further demonstrates the effects of the p38 MAPK pathway on intervertebral disc degeneration, and deepens our understanding of the treatment and prevention of IDD.

## INTRODUCTION

Intervertebral disc degeneration (IDD) is the most common diagnosis in patients with lower back pain. The most common pathological causes of IDD include genetics, age, and inadequate transport of metabolites [1]. Among the dozens of biological causes of IDD, inflammation is known to be one of the most significant. Degenerated discs can display high levels of inflammation, which can lead to stress-induced cell senescence and increased apoptosis [2] and can play a crucial role in disrupting tissue homeostasis. Disruption of homeostasis in the disc allows inflammatory factors to accumulate, and the phagocytic action of macrophages leads to the development of IDD.

It is well known that inflammatory cytokines can activate multiple intracellular signaling cascades, leading to changes in gene expression and ultimately to various corresponding biological effects. The mitogen-activated protein kinase (MAPK) pathway plays an important part in signal transduction cascades regulating inflammation, with many of its downstream substrates regulating the production of inflammatory cytokines and leucocyte migration [3]. The main kinases involved in the MAPK pathway cascade are MAPK kinase kinase (MKKK), MAPK kinase (MKK), and MAPK, which includes four subtypes: ERK, p38 MAPK, c-jun-terminal kinase, and ERK5. Among these subtypes, p38 MAPK is most closely associated with the regulation of inflammatory response [4]. The p38 MAPK pathway is critical for leucocyte migration and activation, TNF-α-mediated expression of e-selectin and vascular cell adhesion molecule, interleukin-8 (IL-8)-mediated upregulation of neutrophils, and production of the inflammatory cytokines TNF-α and IL-1β [3, 5]. In addition, the p38 MAPK pathway can mediate the effects of TNF-α and IL-1β, which include neutrophil activation, cell adhesion changes, cell degranulation, and oxidative bursts, ultimately leading to cell apoptosis [6, 7]. In summary, the p38 MAPK pathway plays a central role in the regulation of various inflammatory response.

Recently, an increasing number of studies have demonstrated the importance of the p38 MAPK pathway in IDD. It has been shown that p38 MAPK expression in nucleus pulposus cells (NPCs) is correlated with IDD in both *in vitro* [8, 9] and *in vivo* [7] studies. In addition, studies have shown that activation of the p38 MAPK pathway actively causes the degeneration of disc tissue. There are several different views on the mechanism underlying this p38 MAPK pathway-induced IDD, but there is a common focus on the pathway’s relationship with inflammatory factors. Some researchers believe that the p38 MAPK pathway increases the production of inflammatory cytokines and promotes the degradation of intervertebral protein polysaccharides, which leads to the degeneration of intervertebral discs [10]. Other researchers believe that the p38 MAPK pathway leads to disc degeneration by inducing excessive cell apoptosis [11]. Activation of the p38 MAPK pathway can also induce macrophages to secrete inflammatory cytokines (e.g., TNF-α, IL-1, and IL-6) [12]. Studer and his colleagues suggested that the p38 MAPK pathway is a potential therapeutic target for delaying the degeneration of NPCs [13].

As a member of the MAPK family, the p38 protein is essentially a protein tyrosine kinase (PTK) and its activation depends on its phosphorylation [4]. Therefore, we hypothesized that a highly effective PTK inhibitor could be used to inhibit the activation of the p38 MAPK pathway, thereby slowing down the degeneration of the intervertebral disc tissue. As a highly effective PTK inhibitor, many studies have shown that Genistein can inhibit p38 MAPK pathway activation in various systems and tissues, resulting in the inhibition of cell apoptosis and inflammatory factor synthesis [14–16]. In this study, we investigated the effect of the PTK inhibitor Genistein in a rat IDD model. We assessed the degeneration of NPCs and other disc tissues at various time points after administering a range of Genistein doses, in order to understand the potential value of Genistein in inhibiting IDD.

## RESULTS

### IL-1β induces degeneration of NPCs

**Figure 1:**
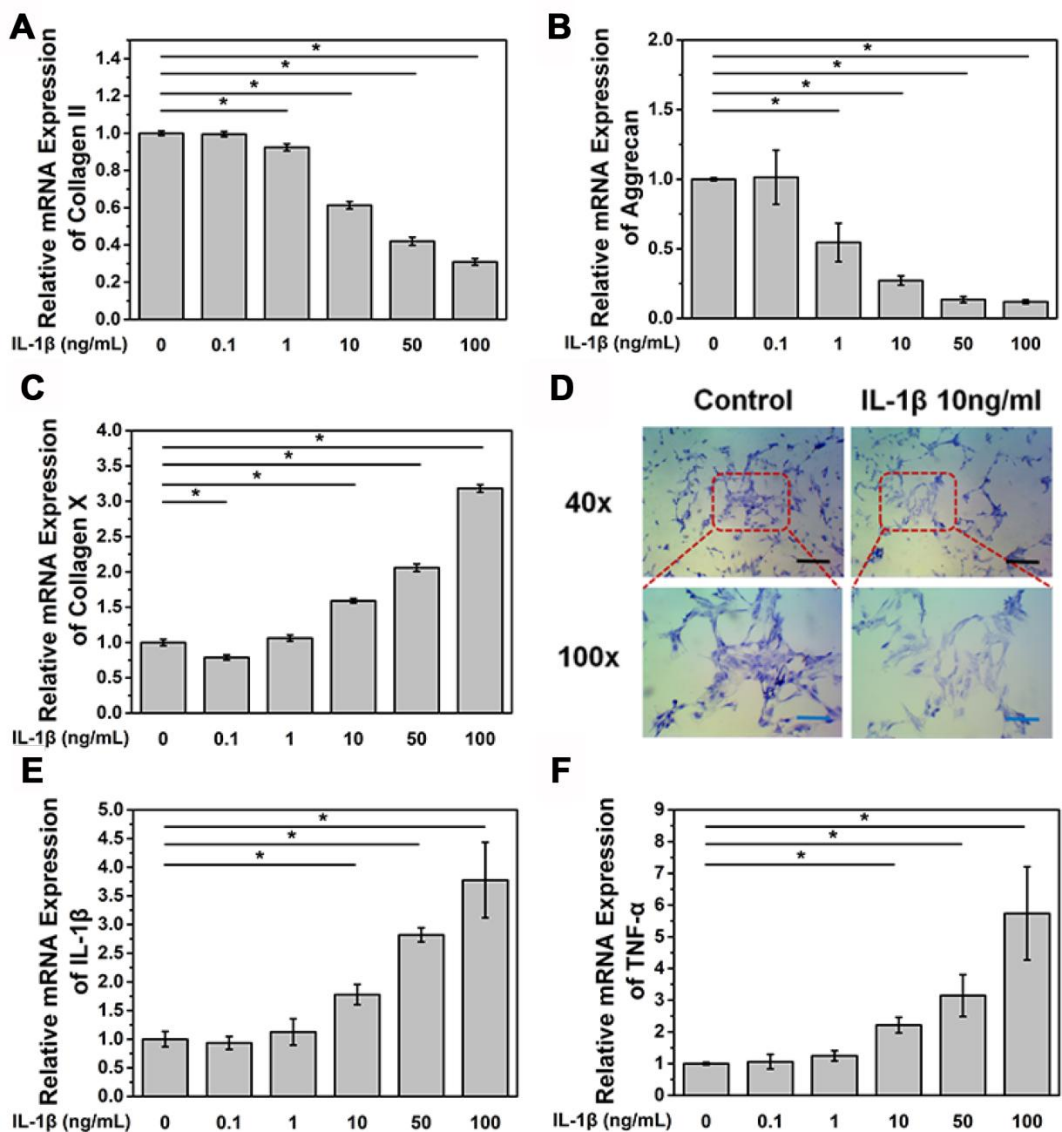
**IL-1β treatment induces NPC degeneration and increases the expression of inflammatory cytokines.** (**A**) mRNA expression of COL2A in NPCs treated with IL-1β. *p<0.05 (**B**) mRNA expression of aggrecan in NPCs treated with IL-1β. *p<0.05 (**C**) mRNA expression of collagen X in NPCs treated with IL-1β. *p<0.05 (**D**) Toluidine blue staining of NPCs treated with IL-1β. Black scale bar represents 100 μm. Blue scale bar represents 25 μm. (**E**) mRNA expression of IL-1β in NPCs treated with IL-1β. IL-1β (**F**) mRNA expression of TNF-α in NPCs treated with IL-1β. *p<0.05.

Primary NPCs were isolated from a male Sprague Dawley (SD) rat and cultured in a 6-well plate (5×10^5^ cells per well). Cells were treated with different concentrations of IL-1β and were collected after 24 h. We found that IL-1β treatment reduced the mRNA expression level of COL2A1, aggrecan, and collagen X in a dose-dependent manner (Figure 1A–1C). The reduction in the mRNA expression of aggrecan and COL2A1 was found after treatment with IL-1β at a concentration of 1 ng/mL (p<0.05, Figure 1A and 1B), While significant reduction in the mRNA expression of collagen X was found after treatment with IL-1β at a concentration of 10 ng/mL (p<0.05, Figure 1C). Toluidine blue staining assay also showed that aggrecan expression decreased after 24 h treatment with IL-1β at a concentration of 10 ng/mL (Figure 1D). In contrast, the mRNA expression of the inflammatory factors IL-1β and TNF-α was higher than the control group after treatment with IL-1β at a concentration of 10 ng/mL (p<0.05, Figure 1E and 1F). Accumulation of many inflammatory factors and the loss of type II collagen and aggrecan are important causes of IDD.

**Figure 2:**
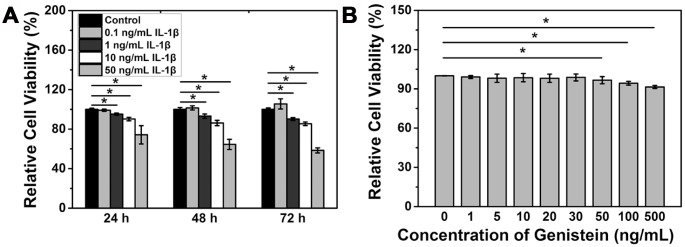
**IL-1β treatment reduces NPC viability.** (**A**) Viability of NPCs treated with IL-1β. *p<0.05 (**B**) Viability of NPCs treated with IL-1β and Genistein. *p<0.05.

Cell counting kit-8 (CCK-8) assay was performed to measure the viability of NPCs after IL-1β treatment. A concentration of IL-1β above 1 ng/mL significantly reduced the viability of NPCs (p<0.05, Figure 2A). The above results indicate that IL-1β can induce degeneration of NPCs.

### Genistein does not affect the cell viability of NPCs

NPCs were treated with different concentrations of Genistein for 24 h and CCK-8 assay was performed to measure the cell viability. The results showed that a low concentration of Genistein does not affect viability of NPCs, while concentrations above 50 ng/mL can inhibit cell proliferation and significantly reduce the viability of NPCs (p<0.05, Figure 2B). Because of this finding, a concentration of Genistein lower than 30 ng/mL was chosen for the subsequent studies.

### Genistein suppresses the degeneration of NPCs *in vitro*

**Figure 3:**
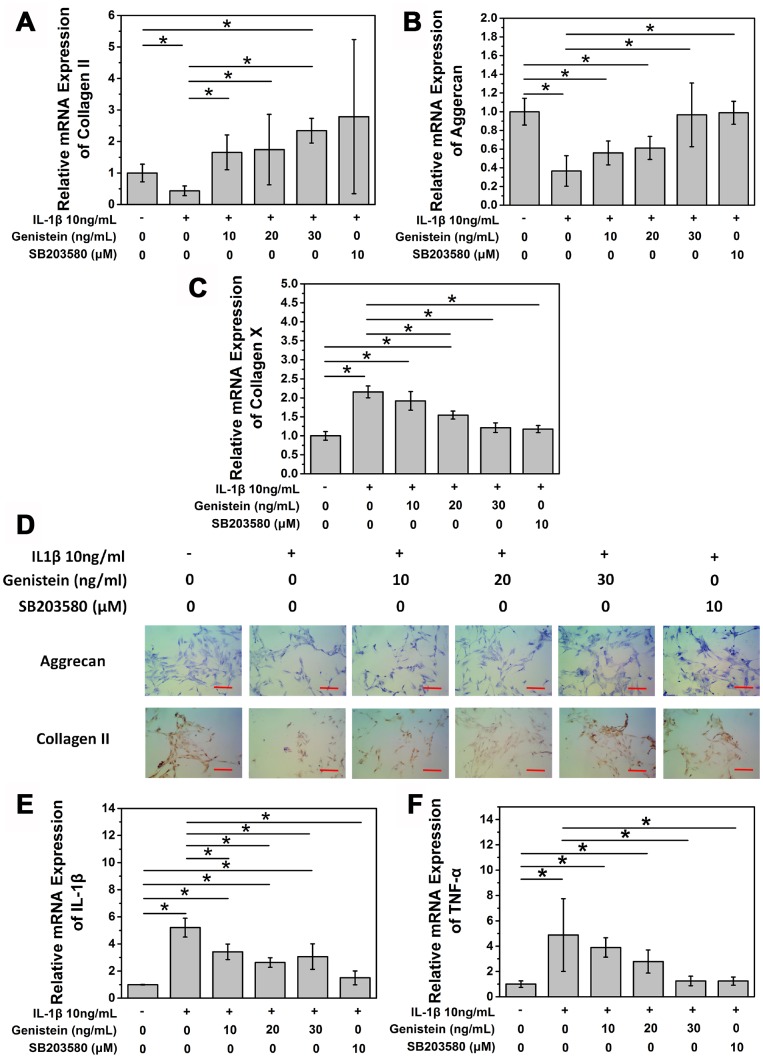
**Genistein suppresses the degeneration of NPCs *in vitro*.** (**A**) mRNA expression of COL2A in NPCs treated with IL-1β and Genistein or SB203580. *p>0.05 (**B**) mRNA expression of aggrecan in NPCs treated with IL-1β and Genistein or SB203580. *p>0.05 (**C**) mRNA expression of collagen X in NPCs treated with IL-1β and Genistein or SB203580. *p>0.05 (**D**) Toluidine blue staining and cell immuohistochemmistry stainingof NPCs treated with IL-1β and Genistein. Red scale bar represents 25 μm (**E**) mRNA expression of TNF-α in NPCs treated with IL-1β and Genistein or SB203580. *p<0.05 (**F**) mRNA expression of IL-1β in NPCs treated with IL-1β and Genistein or SB203580. *p<0.05.

After confirming the degenerative effect of IL-1β on NPCs in our *in vitro* IDD model, we conducted a series of experiments to study the effect of the PTK inhibitor Genistein on IL-1β-induced degeneration. Normal rat NPCs were seeded on a 6-well plate at a density of 5×10^5^ cells/well. The next day, cells were either left untreated (negative control), or stimulated using complete medium containing 10 ng/mL IL-1β for 8 h. Cells were then treated with 0, 10, 20, or 30 ng/mL Genistein for 24 h. As a positive control, 10 μM SB203580, a specific p38 MAPK inhibitor, was used. Compared with the negative control group (no IL-1β treatment), the expression of COL2A1 and aggrecan mRNA in cells treated with IL-1β alone was significantly decreased (p<0.05, Figure 3A and 3B). The SB203580 positive control group after IL-1β treatment showed no difference compared with the negative control group. After treatment with different doses of Genistein, the expression of aggrecan and COL2A1 mRNA showed a dose-dependent increase (Figure 3A and 3B). Low concentrations of Genistein could not completely reverse the influence of IL-1β on aggrecan mRNA expression, but treatment with high concentrations of Genistein showed a much better effect compared to the no Genistein group (p<0.05, Figure 3A). Type II collagen production also improved after Genistein treatment (p<0.05, Figure 3B). Both Genistein and SB203580 showed inhibitory effects on the mRNA expression of collagen X, a degeneration indicator (p<0.05, Figure 3C). To further investigate the effect of Genistein on aggrecan content in cells, we performed toluidine blue staining. After 8 h treatment with 10 ng/mL IL-1β, and 24 h treatment with Genistein (10, 20, or 30 ng/mL), aggrecan content increased in a dose-depended manner (Figure 3D). Cell immunohistochemistry of type II collagen showed a similar trend (Figure 3D). The expression of IL-1β and TNF-α mRNA showed the opposite trend from aggrecan and COL2A (p<0.05, Figure 3E and 3F). Genistein could not completely reverse the influence of IL-1β on the expression of IL-1β (p<0.05, Figure 3E), but at a high concentration could reverse the effect of IL-1β on the expression of TNF-α (p>0.05, Figure 3F). These results indicate that Genistein may play its role by inhibiting the expression of inflammation cytokines.

**Figure 4:**
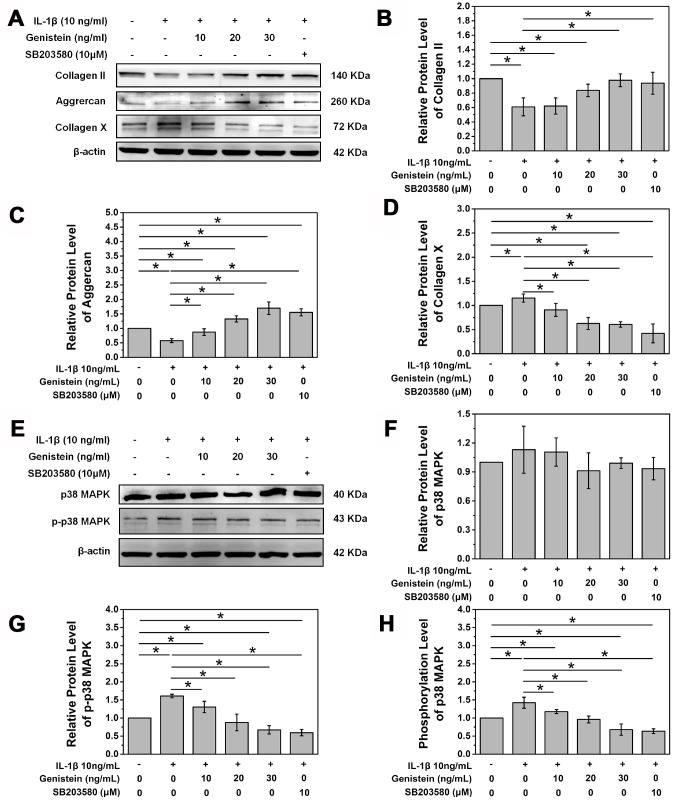
**Genistein and p38 signal pathway inhibitor SB203580 attenuate the degeneration of NPCs and inhibit the phosphorylation of p38.** (**A**) Protein expression of COL2A, aggrecan, and collagen X in NPCs treated with Genistein or SB203580. (**B**) Quantification of COL2A protein expression. *p<0.05 (**C**) Quantification of aggrecan protein expression. *p<0.05 (**D**) Quantification of collagen X protein expression. *p<0.05 (**E**) Protein expression of p38 and p-p38 in NPCs treated with Genistein or SB203580. (**F**) Quantification of p38 MAPK protein expression. *p<0.05 (**G**) Quantification of p-p38 MAPK protein expression. *p<0.05

Western blot further verified the protective effect of Genistein. Genistein reversed the inhibitory effect of IL-1β on type II collagen and aggrecan expression (p<0.05, Figure 4A–4C). At a high concentration, Genistein actually further increased the expression of aggrecan (p<0.05, Figure 4A–4C). We speculate that Genistein might inhibit inflammatory cytokine expression, thereby reducing degradation of aggrecan. Collagen X expression is an indicator of NPC degeneration, and treatment with Genistein significantly inhibited the IL-1β-induced increase in collagen X protein expression (p<0.05, Figure 4A and 4D). These results suggest that Genistein can delay the degeneration of NPCs.

### Effects of Genistein on the p38 MAPK signaling pathway

In order to further study the principle of Genistein in delaying degeneration in rat NPCs after IL-1β treatment, western blotting was performed to examine the protein levels of p38 and p-p38. Rat NPCs were seeded in a 6-well plate, grown to confluency, and then starved overnight in DMEM/F12 medium containing 1% FBS. The next day, cells were switched to serum-free DMEM/F12 culture medium containing 10 ng/mL IL-1β for 8 h and then treated with Genistein (10, 20, or 30 ng/mL) or 10 μM of SB203580. IL-1β treatment showed no effect on the protein expression of p38 MAPK (p>0.05, Figure 4E and 4F), but did significantly increase its phosphorylation (p<0.05, Figure 4E, 4G, and 4H). SB203580, a p38 MAPK pathway inhibitor, significantly inhibited the phosphorylation of p38 MAPK (p<0.05, Figure 4E, 4G, and 4H). Genistein also inhibited the phosphorylation of p38 MAPK in a dose-dependent manner (p<0.05, Figure 4E, 4G, and 4H). Treatment with a high concentration of Genistein was even able to inhibit the intrinsic phosphorylation of p38 MAPK in NPCs (p<0.05, Figure 4E, 4G, and 4H). The above results demonstrate that Genistein plays its protective role in IDD via inhibiting the phosphorylation of p38.

### Genistein affects downstream p38 MAPK signaling

**Figure 5:**
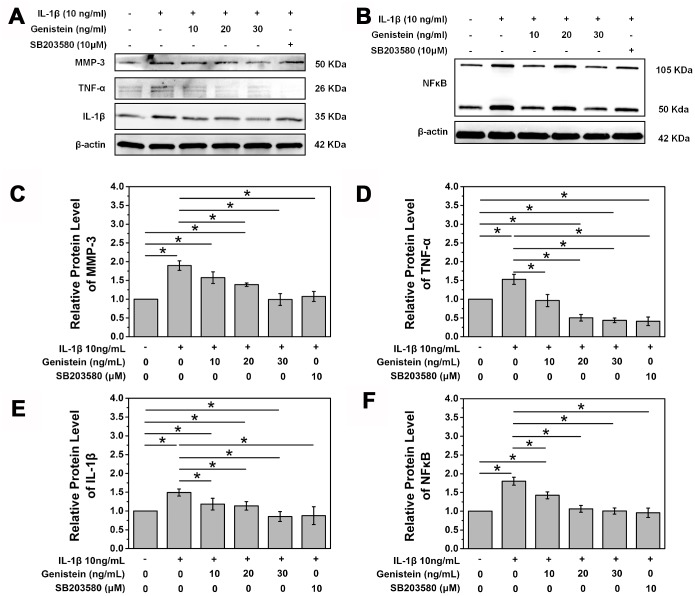
**Genistein inhibits degradation and inflammation of NPCs *in vitro*.** (**A**) Protein expression of MMP-3, IL-1β, and TNF-α in NPCs treated with Genistein or SB203580. (**B**) Protein expression of NF-κB in NPCs treated with Genistein or SB203580. (**C**) Quantification of MMP-3 protein expression. *p<0.05 (**D**) Quantification of TNF-α protein expression. *p<0.05 (**E**) Quantification of IL-1β protein expression. *p<0.05 (**F**) Quantification of NF-κB protein expression. *p<0.05.

We further explored the expression of the downstream classical proteins activated by the p38 MAPK pathway, IL-1β, TNF-α, MMP-3, and NF-κB (Figure 5A and 5B). MMP-3 is a member of the matrix metalloproteinase family and plays a vital role in NPC matrix degradation. Western blot showed that Genistein significantly inhibited the expression of MMP-3, confirming its protective effect (p<0.05, Figure 5A and 5C). Similarly, Genistein significantly inhibited the expression of the inflammatory cytokines IL-1β and TNF-α (Figure 5A, 5D, and 5E). NF-κB is another important inflammatory regulator and its expression was also inhibited by Genistein in a dose-dependent manner (Figure 5B and 5F). These experiments further confirmed the key role of Genistein in delaying the degeneration of intervertebral discs.

### Genistein suppresses the degeneration of NPCs *in vivo*

### MRI results and Thompson score

**Figure 6:**
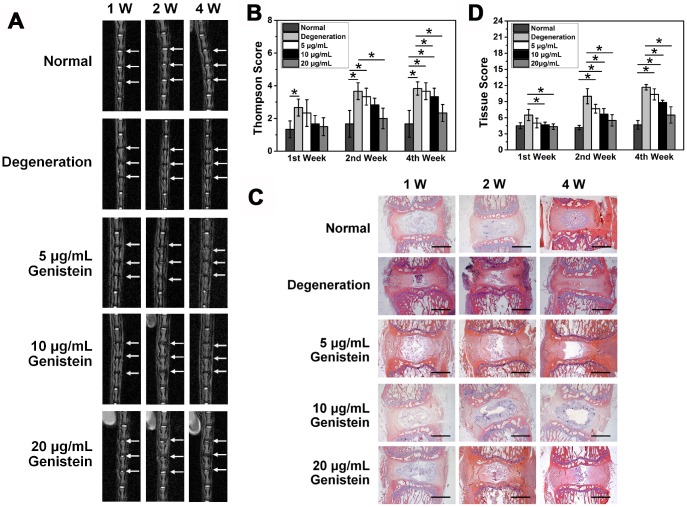
**Genistein suppresses the degeneration of NPCs *in vivo*.** (**A**) MRI images of the caudal intervertebral discs in rats. (**B**) Thompson score of caudal MRI images from rats after surgery and injection with Genistein. (**C**) HE staining of intervertebral disc sections from rats after surgery and injection with Genistein. Black scale bar represents 1 mm. (**D**) Histology score of intervertebral disc sections.

A total of 30 clean SD rats (male, weighing 400±20 g) without any congenital malformations or disc degeneration of the caudal vertebrae were randomly divided by the digital table method into 5 groups: normal group, degeneration (sham) group, and 3 experimental groups, each containing 6 rats. The rats in the degeneration (sham) group and the 3 experimental groups all underwent surgery to puncture their intervertebral discs. Rats in the sham group received no treatment, while rats in the experimental groups were given different concentrations of Genistein. The experimental groups showed varying levels of improvement in the intervertebral disc signal compared with the degeneration group one week after surgery. MRI results showed that treatment with the two higher Genistein doses (10 μg/mL and 20 μg/mL) resulted in a high signal one week after injection. However, the high signal area of the 20 μg/mL treatment group was much larger than that of the 10 μg/mL treatment group. With increasing time from the injection date, the intervertebral disc signal in the experimental groups decreased. Four weeks after intervention, the intervertebral discs in the 5 μg/mL and 10 μg/mL treatment groups showed a low signal level, and the spinal space became narrower in the 5 μg/mL treatment group, while some high signal intensity was still observed in the 20 μg/mL treatment group. The intervertebral disc signal strength showed a positive correlation with the Genistein dose but decreased gradually as a function of time (Figure 6A).

One week after drug intervention, there was no statistically significant difference in intervertebral disc signal between the 5 μg/mL treatment group and the degeneration group (Thompson score, p>0.05). However, both the 10 μg/mL and 20 μg/mL treatment groups were significantly different from the degeneration group (p< 0.05). Four weeks after drug intervention, the difference remained statistically significant in the 20 μg/mL treatment group compared to the degeneration group (p<0.05) (Figure 6B).

### HE staining and histological scores

One week after treatment with 5 μg/mL Genistein, the height and volume of the intervertebral space and the number of chondroid nucleus pulposus all decreased, and the annulus fibrosus displayed varying degrees of distortion and damage. The degeneration was evident. However, the boundary between the nucleus pulposus and the annulus fibrosus remained clear. Four weeks after treatment with 5 μg/mL Genistein, the nucleus pulposus tissue was completely lost, and the annulus fibrosus damage was further aggravated. One week after treatment with 10 μg/mL or 20 μg/mL Genistein, more chondroid nucleus pulposus were observed, and with a neater arrangement compared to the 5 μg/mL Genistein group at the same time point. The annulus fibrosus displayed a regular ring structure and each layer was arranged neatly without significant degeneration. After four weeks, both the 10 μg/mL and 20 μg/mL treatment groups displayed degeneration, as evidenced by reduction in NPCs and distortion of the annulus fibrosus. However, the 20 μg/ml treatment group showed less degeneration than the 10 μg/ml treatment group (Figure 6C).

We developed a scoring system to quantitively assess the histology results (Table 1) [17]. The histological score of the 5 μg/ml treatment group was not statistically significant at any of the time points compared with the degeneration group (p>0.05, Figure 6D), but the histological scores of both the 10 μg/ml and 20 μg/ml treatment groups were statistically significant after one week (p<0.05, Figure 6D). There was no statistical difference in the 10 μg/ml group compared with the degeneration group four weeks after treatment, whereas the difference was still statistically significant in the 20 μg/ml treatment group (p<0.05, Figure 6D). The results of HE staining were consistent with the imaging results, which provides histological evidence that Genistein can delay the progression of IDD.

**Table 1:** Grading for morphology

		**Morphology change under optical microscope**	**Grade**
I	Annulus Fibrosus (AF)	Normal texture and free of damage and distortion	1
		The damaged and distortion area is less than 30%	2
		The damaged and distortion area is more than 30%	3
II	Boundary between AF and NP	Normal	1
		Micro disrupted	2
		Medium or severe disrupted	3
III	NP Cells	Normal cells with large amounts of vacuoles	1
		Cells and vacuoles decreased slightly	2
		Cells decreased moderately or severely without vacuoles	3
IV	NP Matrix	Normal gel appearance	1
		Slightly congealed	2
		Moderate or severe condensation	3

### Immunohistochemical results

**Figure 7:**
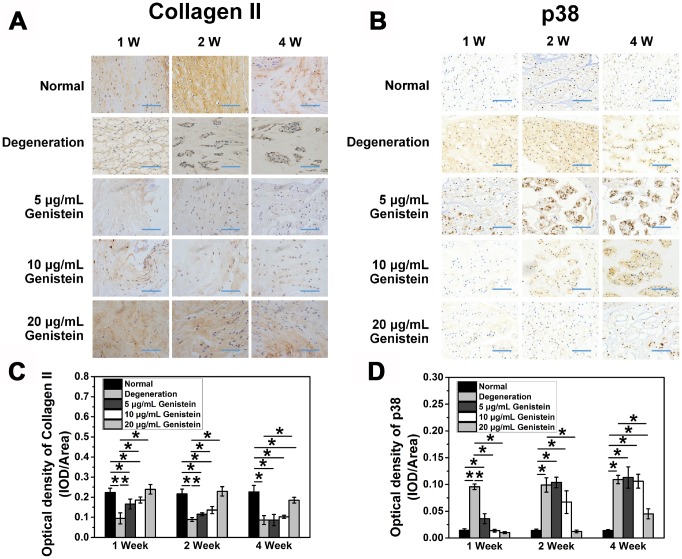
**Genistein attenuates the degeneration and inflammation of NPCs *in vivo*.** (**A**) COL2A expression in intervertebral disc sections from rats after surgery and injection with Genistein. *p<0.05 (**B**) p38 expression in intervertebral disc sections from rats after surgery and injection with Genistein. *p<0.05 (**C**) Optical density quantification of COL2A. Blue scale bar represents 100 μm. (**D**) Optical density quantification of p38. Blue scale bar represents 100 μm.

Immunohistochemical staining of type II collagen and p38 was performed to identify the expression of these two key proteins *in vivo* (Figure 7A and 7B). One week after treatment with 5 μg/ml Genistein, the type II collagen staining in the nucleus pulposus was light yellow, and the color gradually faded with time. Four weeks later, the staining was essentially negative. One week after treatment with 10 μg/mL and 20 μg/mL Genistein, a higher degree of yellow or brown positive staining was observed in the nucleus pulposus. Two weeks after treatment, the positive staining in the 10 μg/mL treatment group began to fade. However, the positive staining in the 20 μg/mL treatment group displayed a slight reduction in area only after four weeks. The staining gradually decreased over time in all treatment groups. The protective effect of Genistein on type II collagen levels was dose-dependent, with the 10 μg/mL and 5 μg/mL treatment groups displaying intermediate and low staining intensity, respectively (Figure 7A). Quantitative analysis of immunohisto-chemical sections also showed the significant protective effect of Genistein (p<0.05, Figure 7C).

The immunohistochemical staining of p38 showed the opposite result. Treatment with a low concentration of Genistein (5 μg/mL or 10 μg/mL) did not reverse the increased expression of p38 but did significantly delay the time point of expression (p<0.05, Figure 7D). Treatment with a high concentration of Genistein (20 μg/mL) maintained the normal expression of p38 for the first two weeks, a slight but significant increase in p38 was detected after four weeks (p<0.05, Figure 7D). These results further validate that Genistein functions to delay IDD via inhibiting the p38 MAPK pathway.

## DISCUSSION

Various sources of stimulation such as growth factors, inflammatory cytokines, or various environmental stresses can activate p38 MAPK. Many studies have shown that IL-1β is an important stimulant for the p38 MAPK pathway [18–21]. Typical activation of the p38 MAPK pathway is via the MAPK mediated phosphorylation of p38 MAPK on the activation ring [4]. As p38 MAPK activity is mainly regulated by phosphorylation and dephosphorylation, inhibiting its phosphorylation is the key to decreasing p38 MAPK activation, and thereby delaying the degeneration of intervertebral disc tissue.

One group of specific p38 MAPK inhibitors is the synthetic pyridimidazole derivatives, of which SB203580 is an example. Morel et al. found that rather than blocking the effect of upstream signaling on p38 activation, SB203580 directly blocked ATP binding to the active site of p38 (Thr106), subsequently inactivating the kinase [22]. The effect of SB203580 was found to be relatively stable. Several studies have tried to inhibit the degeneration of intervertebral disc cells using these p38 pathway inhibitors *in vitro* [7, 23], but *in vivo* experiments have not previously been attempted. As they are synthetic substances, it is unknown whether pyridimidazole derivatives would have toxic effects on humans.

Genistein is a natural flavonoid compound that is primarily derived from legumes and has been widely used in the prevention and treatment of tumors [24, 25], diabetes [26, 27], skin diseases [28], and cardiovascular diseases [29]. As early as 1987, Akiyama et al. discovered that Genistein is a highly effective inhibitor of PTKs [30, 31], which mediate their effects mainly through ATP competition. As a subtype of MAPK, the p38 protein is itself a PTK. Previous studies have shown that Genistein can inhibit activation of the p38 MAPK pathway in different systems and tissues [14–16], leading to inhibition of apoptosis and synthesis of inflammatory factor. Therefore, it seemed logical to explore whether Genistein could also inhibit the degeneration of the nucleus pulposus.

It is well known that intervertebral disc tissue undergoes various biochemical and morphological changes during aging and degeneration, including the loss of proteoglycans, water, and type II collagen [32]. First, we activated the p38 MAPK pathway using the inflammatory factor IL-1β at the excessive concentration of 10 ng/mL to induce disc degeneration. We found that IL-1β had a significant positive effect on the proliferation of rat NPCs, consistent with the results of previous studies [33, 34]. In addition, the mRNA levels of COL2A1 and aggrecan were significantly lower in the treatment group than in the control group, whereas levels of IL-1β and TNF-α were significantly higher. With an increase in IL-1β treatment time, this effect continued to increase and stabilize. Toluidine blue staining and cell immunohistochemistry also showed that the expression of COL2A1 and aggrecan was significantly lower after IL-1β stimulation compared to the control group. Western blotting results showed that the ratio of p-p38 to p38 protein after IL-1β treatment was higher than in the control group, but this did not change significantly with time. The time course illustrated that p38 was intensely and rapidly phosphorylated after IL-1β treatment, reaching its peak within 15 min. Therefore, IL-1β can rapidly lead to a steady activation state of the p38 MAPK pathway, eventually accelerating the degeneration of rat NPCs.

After addition of the PTK inhibitor Genistein, the mRNA level of COL2A1 and aggrecan was significantly increased compared to treatment with IL-1β alone, and this effect increased and became more stable in a dose-dependent manner. Immunohistochemistry and toluidine blue staining also showed a slight increase in type 2 collagen levels. The addition of Genistein clearly inhibited the degeneration of intervertebral disc cells. However, the mRNA level of IL-1β and TNF-α was still higher than in the control group, indicating that Genistein cannot reverse the synthesis and secretion of inflammatory cytokines. The mechanism for increasing the expression of COL2A1 and aggrecan may therefore not be related to blocking the inflammatory environment. Disc tissue degeneration was likely still in progress due to the persistence of inflammatory cytokines. Our results therefore suggest that Genistein cannot reverse disc degeneration, but it can decelerate the process.

Our research further demonstrated that Genistein is able to inhibit the p38 MAPK pathway. Western blotting results showed that Genistein reduced the phosphorylation of p38 compared to the IL-1β only control group, and even enhanced the dephosphorylation of p38 compared to control, which is indicative of a substantial inhibitory effect on the p38 MAPK pathway. Both the 10 ng/mL and 20 ng/mL doses of Genistein were sufficient to effectively execute its PTK inhibitor function, leading to dephosphorylation of p-p38. This dephosphorylation effect was comparable to that of the specific inhibitor SB203580 at a concentration of 10 μM.

Animal experiments further demonstrated that Genistein can slow down the progression of disc degeneration. The effective improvement in IDD was positively correlated to the dose of Genistein. However, combined with the medical imaging results and histological assessments, even the 20 μg/mL group still showed mild degeneration, suggesting that Genistein was not able to completely prevent the occurrence of disc degeneration, consistent with the results of our *in vitro* experiments. The type II collagen staining further supports our hypothesis that Genistein can delay the progression of disc degeneration. More importantly, all animals survived without significant acute or chronic side effects. Considering that Genistein is derived from natural sources, its application in a clinical setting could be more extensive than synthetic inhibitors, which may have unwanted effects.

Despite the importance of our findings, there are still some limitations to this study. First, we only administered a single injection of the PTK inhibitor Genistein into the intervertebral disc. Whether the degeneration observed four weeks after drug intervention was due to drug metabolism is beyond the scope of this study. A follow-up study should be performed with the administration of multiple Genistein injections over time to assess whether this could enhance its therapeutic effect. In addition, we only used a single PTK inhibitor, Genistein, in this study. Whether other PTK inhibitors can achieve the same effect is also worth investigating in the future.

In summary, we demonstrated the important role of Genistein, a highly effective PTK inhibitor, in inhibiting the p38 MAPK pathway and delaying the degeneration of the intervertebral disc. Genistein reduces p38 phosphorylation, blocks p38 MAPK pathway activation, and reduces the corresponding inflammatory response, thus playing a key role in inhibiting IDD. These findings reveal new biological functions of Genistein, further demonstrate the effects of the p38 MAPK pathway on intervertebral disc degeneration, and deepen our understanding of the treatment and prevention of IDD.

## MATERIALS AND METHODS

### Isolation and culture of primary NPCs

A male Sprague Dawley (SD) rat, provided by the Animal Experimental Center of Soochow University, weighing 225-250 g was sacrificed and then disinfected in 75 % alcohol for 15 min. The root of the tail (including the caudal vertebral disc tissue) was cut under sterile conditions and peeled to fully expose the tail intervertebral disc. The disc was then moved to a glass plate containing 2 x penicillin-streptomycin in PBS for 5 min. The intervertebral disc fiber ring was cut using a sterile sharp surgical blade, and the nucleus pulposus tissue was completely removed and placed in PBS containing 100 x penicillin-streptomycin. The tissue was cut into 1 mm^3^ pieces and moved to individual sterile centrifuge tubes. Type II collagenase solution (0.2 %, Thermo Fisher Scientific) was added at a 5:1 volume ratio to the tissue, followed by incubation in a water bath at 37 °C for 2 h with gentle shaking every 20 min to digest the tissue. The digestion was neutralized by addition of an equal volume of DMEM/F12 complete culture medium, followed by centrifugation at 1000 rpm for 5-8 min. The supernatant was aspirated and the cells were resuspended in fresh DMEM/F12 complete culture medium and transferred to a culture bottle. The cells were cultured in a saturated humidity incubator with a constant temperature of 37 °C and 5% CO_2_. All the experimental procedures were performed at the First Affiliated Hospital of Soochow University. All the experimental procedures were approved by the Ethics Committee of the First Affiliated Hospital of Soochow University and were carried out in strict accordance with Declaration of Helsinki (1964) and the Laboratory Animal Guidelines for Ethical Review of Animal Welfare (GB/T 35892-2018, China).

### Reverse transcription–polymerase chain reaction

The NPCs were lysed in Trizol (Ambion, TX, USA) following the manufacturer’s protocol. Total RNA was reverse transcribed to cDNA using RevertAid RT Reverse Transcription Kit (Thermo Scientific, CA, USA) according to the manufacturer’s protocol. Quantitative PCR (qPCR) was performed using SYBR Green Master mix reagent (Toneker, Shanghai, China) according to the manufacturer’s protocol to measure the mRNA level of IL-1β, TNF-α, COL2A1, and aggrecan. Primer sequences are shown in Table 2. The experiment was repeated three times independently.

**Table 2:** Primer sequences for reverse transcription–polymerase chain reaction.

GAPDH-F	TTCAACGGCACAGTCAAG
GAPDH-R	TGGTTCACACCCATCACA
IL-1β-F	CTGTGACTCGTGGGATGATG
IL-1β-R	GGGATTTTGTCGTTGCTTGT
TNF-α-F	AGATGTGGAACTGGCAGAGG
TNF-α-R	CCCATTTGGGAACTTCTCCT
Collagen X-F	CCCTTTTTGCTGCTAGTATCC
Collagen X-R	CTGTTGTCCAGGTTTTCCTGGCAC
COL2A1-F	TCACGTACACTGCCCTGAAG
COL2A1-R	TGTCCACACCAAATTCCTGA
Aggrecan-F	CAACCTCCTGGGTGTAAGGA
Aggrecan-R	TGTCCACACCAAATTCCTGA

### Toluidine blue staining

Primary NPCs were treated with different concentrations of IL-1β and/or Genistein for the indicated time. Cells were then fixed with 4% paraformaldehyde (PFA) for 15-20 min and stained with toluidine blue dye (Sigma-Aldrich, MO, USA) for 2-4 h. The remaining dye was washed off with 95 % ethanol, then cells were rinsed with PBS, sealed, and observed by microscopy.

### Cell immunohistochemistry

Primary NPCs were treated with different concentrations of IL-1β and/or Genistein for the indicated time. Cells were then fixed with 4% PFA for 15-20 min and 3 % H_2_O_2_ was added for 10 min. Cells were blocked with goat serum (Abcam, CA, USA) for 20 min, followed by incubation with the primary antibody (anti-collagen II, Abcam) at 4 °C overnight. The next day samples were incubated with HRP-conjugated second antibody (Bioworld, CA, USA) at 37 °C for 40 min. DAB was added and the staining was observed under a microscope. Where hematoxylin was used for nuclear staining, this was followed by dehydration with 1% hydrochloric acid in ethanol, a PBS wash, then sealing and microscope examination.

### Cell counting kit-8 assay

NPCs were seeded in a 96-well plate at 5000 cells per well. After 24 h, the cells were treated with different concentration of IL-1β or Genistein. CCK-8 (Dojindo Molecular Technologies, Inc, Japan) assay was used for measuring cell viability following the manufacturer’s protocol. The supernatant was measured with an absorbance plate reader (Spectra Max M5, Molecular Device, CA, USA) at an OD value of 450 nm.

### Western blots

Cells were harvested and lysed on ice using 100 μL RIPA buffer (Thermo Fisher Scientific, CA, USA) containing Phenylmethylsulfonyl fluoride. After separation on 10% or 5% polyacrylamide gels, proteins were transferred to polyvinylidene difluoride (PVDF) membrane (Millipore, MA, USA). The membranes were blocked with 5% non-fat milk for 1 h at room temperature and then incubated with different primary antibodies (collagen II, aggrecan, and collagen X were from Novus, CO, USA; p38 MAPK was from Boster Biological Technology, CA, USA; p-p38 MAPK was from Santa Cruz, CA, USA; MMP-3, IL-1β, TNF-α, and NF-κB were from Abcam, CA, USA) overnight at 4 °C. The next day membranes were incubated with HRP-linked secondary antibodies (goat anti-rabbit and goat anti-mouse were from Beijing Zhongshan Jinqiao Biotechnology) for 1 h at room temperature. The protein signal was detected using an ECL chemiluminescence kit (Thermo Scientific CA, USA) according to the manufacturer’s instructions and observed using a gel image analyzer. The relative protein expression was compared to the control group. The phosphorylation level of p38 MAPK is given as the ratio of phosphorylated p38 MAPK to total p38 MAPK protein.

### Establishment of animal models

A total of 30 clean SD rats (male, weighing 400±20 g) were provided by the Animal Experimental Center of Soochow University. After magnetic resonance scanning and x-ray examination to confirm the absence of any congenital malformations or disc degeneration of the caudal vertebrae, the rats were randomly divided by the digital table method into 5 groups: normal group, degeneration (sham) group, and 3 experimental groups, each with 6 rats. Preoperative x-rays were used to position the Co 7/8, Co 8/9, and Co 9/10 vertebral gaps. The rats were weighed, then intraperitoneally anesthetized with 10% chloral hydrate (3.5 mL/kg). The rats were fixed onto the operating table, and 18G injection needles were used to pierce the Co 7/8, Co 8/9, and Co 9/10 vertebral gaps. The needle tips were inserted perpendicular to the rat tail until complete penetration to the opposite side, then rotated 360° and retracted after 30 s. After surgery, rats were allowed free movement in the cage with no dietary restriction and were closely observed for the presence of urinary retention and infection.

After confirming the establishment of IVD model one week after puncture, rats in the experimental group were injected with 2 μL drugs. The degeneration, or sham, group was punctured and given no further treatment. The experimental groups were divided according to the injection dose of Genistein (5 μg/mL, 10 μg/mL, or 20 μg/mL). Before disc injection, the rat tails were dissected at the Co 7/8, Co 8/9, and Co 9/10 positions and the distance between the skin and the intervertebral core, approximately 8 mm, was measured.

All the experimental procedures were performed at the First Affiliated Hospital of Soochow University. All the experimental procedures were approved by the Ethics Committee of the First Affiliated Hospital of Soochow University and were carried out in strict accordance with Declaration of Helsinki (1964) and the Laboratory Animal Guidelines for Ethical Review of Animal Welfare (GB/T 35892-2018, China).

### MRI examination

Two rats per experimental group were randomly selected 1, 2, and 4 weeks after surgery. Rats were mercy sacrificed and then scanned through MRI. Intervertebral disc signals were obtained on 1.5T Magnetic Resonance (MR) scanner (Philips Eclipse, Germany), using the following parameters of T2-weighted sagittal plane: TR/TE: 3500/102 ms, FOV: 15.0, thickness: 3 mm, interval: 0 mm. The degree of disc degeneration was semi-quantitively assessed by signal intensity on T2-weight image (T2WI) of intervertebral disc and graded with a four-grade modified Thompson system: grade 1: normal; grade 2: slightly decreased signals and obvious reduced high-signal areas; grade 3: moderately decreased signals; grade 4: significantly reduced signals.

### Histological examination

After the imaging examination was completed, the Co 7/8, Co 8/9, and Co 9/10 discs were entirely extracted and subjected to fixation with 10% neutral formaldehyde at room temperature for 24 h. The discs were decalcified in 10% EDTA for 2 weeks and then sliced into 5 horizontal sections 4 μm thick. Slices were dewaxed in xylene twice for 5 mins, dehydrated in graded ethanol (100%, 2 mins; 95%, 1 min; 80%, 1 min; 75%, 1 min), and stained with hematoxylin for 5 mins and eosin for 2 mins. The morphology of the intervertebral disc was observed and scored under a light microscope according to Table 1.

### Immunohistochemical staining

The expression of collagen II and p38 was detected using immunohistochemistry. The slices were dewaxed in xylene, dehydrated in graded ethanol, and incubated in 3% H_2_O_2_ at 37 °C for 10 min. Sections were then washed in PBS, boiled in 0.01M citric acid buffer for antigen retrieval (95 °C, 15-20 min), and blocked in goat serum for 10 mins at 37 °C. Slices were then incubated with primary antibody (anti-collagen II, Abcam; anti-p38 MAPK, Thermo Fisher Scientific) at 4 °C overnight and biotin-conjugated secondary antibody (Bioworld) for 30 mins at 37 °C. Slices were counterstained with hematoxylin and observed under a light microscope.

### Statistical analysis

All quantitative data are presented as mean ± S.D. For parametric data, statistical analyses were performed by one-way ANOVA. For non-parametric data (MRI and histological classification), the Kruskal-Wallis test and Mann-Whitney U test were performed. Differences with values of p < 0.05 were considered statistically significant.
